# Real and Simulated Microgravity: Focus on Mammalian Extracellular Matrix

**DOI:** 10.3390/life12091343

**Published:** 2022-08-29

**Authors:** Elena Andreeva, Diana Matveeva, Olga Zhidkova, Ivan Zhivodernikov, Oleg Kotov, Ludmila Buravkova

**Affiliations:** Department of Cell Physiology, Institute of Biomedical Problems, Russian Academy of Sciences, Khoroshevskoye Shosse 76a, 123007 Moscow, Russia

**Keywords:** mechanoreception, gravireception, microgravity, simulation, extracellular matrix

## Abstract

The lack of gravitational loading is a pivotal risk factor during space flights. Biomedical studies indicate that because of the prolonged effect of microgravity, humans experience bone mass loss, muscle atrophy, cardiovascular insufficiency, and sensory motor coordination disorders. These findings demonstrate the essential role of gravity in human health quality. The physiological and pathophysiological mechanisms of an acute response to microgravity at various levels (molecular, cellular, tissue, and physiological) and subsequent adaptation are intensively studied. Under the permanent gravity of the Earth, multicellular organisms have developed a multi-component tissue mechanosensitive system which includes cellular (nucleo- and cytoskeleton) and extracellular (extracellular matrix, ECM) “mechanosensory” elements. These compartments are coordinated due to specialized integrin-based protein complexes, forming a distinctive mechanosensitive unit. Under the lack of continuous gravitational loading, this unit becomes a substrate for adaptation processes, acting as a gravisensitive unit. Since the space flight conditions limit large-scale research in space, simulation models on Earth are of particular importance for elucidating the mechanisms that provide a response to microgravity. This review describes current state of art concerning mammalian ECM as a gravisensitive unit component under real and simulated microgravity and discusses the directions of further research in this field.

## 1. Introduction

The extracellular matrix (ECM) is a constituent of all tissues, representing an integrating network that consists of specific and similar attributes. The ECM always includes collagen and noncollagen proteins, glycoproteins, and proteoglycans [1,2]. The molecular composition of these structures demonstrates a huge diversity determined by the functional needs of a particular tissue. Meanwhile, it is the structural uniformity that ensures the operation of the ECM as a putative gravity detector. It is reasonable to assume that ECM components can act as an extracellular “mechanical sensor”.

To date, the ECM as a complementary counterpart of gravireception has been studied to a lesser extent than the cellular one. At the same time, the data from space flights and ground-based simulations clearly demonstrate that the skeleton with a well-represented ECM is most sensitive to the lack of gravitational loading [3,4,5]. It is obvious that further progress in the elucidation of the mechanisms of physiological adaptation to microgravity requires the analysis of gravisensitive unit components with a special focus on the involvement of ECM structures.

## 2. Cells in the Local Microenvironment: Functional Unit of Mechanoreception

*Terrestrial Gravity*: The physiological homeostasis of tissues and organs of multicellular organisms is the result of the interaction of cellular and noncellular components that form tissue-specific local milieu. It is believed that a tissue niche is an ensemble that includes cells and the cell secretion products: the ECM and soluble and matrix-deposited biologically active mediators [6]. Cells and matrix components are combined into a unified system by specialized transmembrane structures [7]. Tissue-specific cell populations, various combinations of ECM molecules and soluble and bound mediators create all the diversity of tissues that exist in the body. The homeostasis of such a structural and functional unit is regulated by a bidirectionally controlled feedback loop of cellular (nucleus and cytoskeleton) and extracellular (ECM) elements [8]. The physical properties of the ECM and its molecular composition influence cell migration, proliferation, and differentiation [9,10]. The niche cells continuously produce ECM molecules and enzymes involved in ECM remodeling or degradation, affecting the composition and topography of the surrounding ECM, thus closing the feedback loop. Under gravitation of the Earth, multicellular organisms have formed a mechanism for perceiving external mechanical stimuli (compression, tension, twisting), which includes all niche components [11].

**Figure 1:**
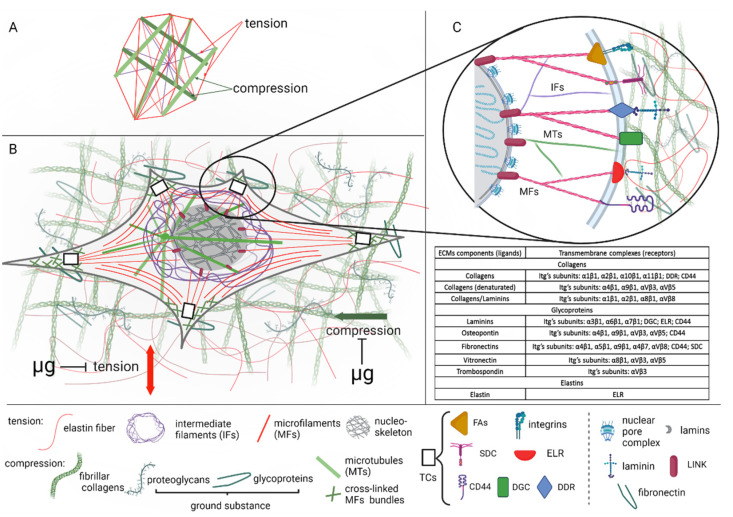
The functional unit of mechanoreception: cellular and extracellular compartments. (**A**) Ingber’s tensegrity model. (**B**) Cellular and extracellular mechanosensitive patterns. Tensegrity stabilizing elements (tension and compression) are modulated by microgravity (µg). (**C**) Interconnection between extracellular and cellular mechanosensitive compartments. Transmembrane complexes (TCs): FAs—focal adhesion complexes; DGC—dystrophin glycoprotein complexes; DDR—discoidin domain receptors; ELR—elastin–laminin receptors; SDC—syndecans; CD44—hyaluronan receptors; LINC—the linker of nucleoskeleton and cytoskeleton. Table inset—extracellular ligands and its receptors. (**A**–**C**) Compression-resistant elements are colored in green. Tensional elements are colored in red. (Created with Biorender.com, https://biorender.com/ (accessed on 10 August 2022).

According to Ingber’s tensional integrity (tensegrity) model, microtubules and cross-linked microfilament bundles form a rigid framework, which ensures cellular resistance against compression [12] (Figure 1A). The plasticity of this framework upon alteration of the external environment is provided by tension-responsible single microfilaments and intermediate filaments tightly interconnected with compression-resistant structures presented by actin fibers and microtubules. Such an integrated cytoskeleton is the intracellular recipient of external mechanical influences, with the signal transmitted to the nuclear lamina and then to the nucleoskeleton [13,14] (Figure 1B). The ECM has its own structures to provide tensegrity. Reticulin and elastin fibrils act as tension elements, and collagen fibers as well as the ground substance act as compression resistance elements. The intracellular and extracellular tensegrity compartments interact using transmembrane focal adhesion complexes recognized as the principal element that transmits information from the ECM to the cytoskeleton (Figure 1B). The conversion of mechanical stimuli from the extracellular to intra-nuclear compartments is executed through highly specialized molecular hubs [15]. The interaction of cytoskeleton and ECM elements is carried out through transmembrane adhesion sites (Figure 1C, Table inset). Focal adhesion (FA) complexes built by the heterodimeric cell surface receptors, integrins, considered as principal molecules, mediated ECM–actin cytoskeleton interaction. Integrin dimers outside of cells are connected with ECM structures, while their transmembrane domains are linked to intracellular actin stress fibers. External stimuli induce cell–matrix adhesion and subsequent signaling cascades evolving tyrosine kinase (FAK) phosphorylation to support cytoskeletal rearrangement and further intracellular transduction of stimuli into the nucleus [16]. In addition, there are the complexes of other receptors executing cell–ECM communication. Dystrophin glycoprotein complexes (DGC) link laminins with actin filaments through dystrophin [17]. Discoidin domain receptors (DDR) are known to bind collagens to myosin–actin complexes [17]. Elastin–laminin receptors (ELR) mediate interplay between laminin/elastin and a-tubulin/F-actin [18]. Syndecans (SDC) connect fibronectins with actin microfilaments [19]. Hyaluronan receptors (CD44) contain binding sites for collagens, laminins, and fibronectins and link these ECM structures with actin cytoskeleton [20].

Inside the cells, the transmission of mechanical stimuli is executed through the cytoskeletal filaments to the specialized structure in the nuclear membrane-linker of the nucleoskeleton and cytoskeleton (LINC) complex, that provides a functional link between the cytoplasmic and nuclear (lamin) network [21] (Figure 1C). Interconnected cellular and extracellular compartments form a mechanosensitive unit, which jointly provides a response to mechanical loading, converting mechanical stimuli into biochemical signals provided by signaling cascades known as mechanotransduction [22,23,24,25]. Under microgravity, the mechanosensitive unit has to adapt to the reduced gravitational loading, reacting as a gravisensitive unit.

*Microgravity*: Over the past decades, real and simulated microgravity studies provided significant progress in the elucidation of the cellular shoulder of the gravisensitive unit response. Under microgravity, cells have been shown to develop numerous, often reversible morphological and functional changes, including cytoskeletal element remodeling, changes in gene expression, and mosaic rearrangement of intracellular regulation systems, which has been detailed in several reviews [26,27,28,29,30]. Alterations in the shape, size, and adhesive properties associated with microtubule and F-actin reorganization have been shown in a variety of cell types [28,30,31,32,33]. These perturbations are based on a significant shift in the transcriptional and translational activity of cytoskeleton-associated genes and molecules, respectively [34,35].

The molecular mechanisms of alterations in cellular tensegrity have not been fully investigated. The gravity-dependent reorganization of tubulin structures is assumed to be regulated by the microtubule organizing center [32,36,37]. A decreased expression of actin and actin-associated proteins, namely Arp2/3 and RhoA, under microgravity may result in the actin cytoskeleton disruption [32,37,38,39]. Opposing views are also available, indicating an increased F-actin level and stress fibril formation resulting in the formation of lamellipodia protrusions [40]. The actin cytoskeleton is considered as the intracellular effector of mechanical stimuli perception from transmembrane integrin complexes [14].

The apparent contradictions in the findings on the cytoskeleton may be explained by time-dependent changes in the cytoskeleton components under real or simulated microgravity. Upon microgravity simulation, F-actin redistribution to the cellular periphery and a decrease in the stress fibril volume were demonstrated as early as after 30 min of exposure. In addition, changes in cytoskeletal gene expression which occurred rapidly were transient and correlated with the cytoskeleton rearrangements [41]. After 120 h, cells partially or completely re-established the actin cytoskeleton microarchitecture. Such restoration upon a long-term simulated microgravity appear to reflect the process of adaptation to the altered gravitational parameters. Interestingly, no pronounced disturbances of microtubule structures were observed at the mentioned time points [10]. The above may indicate that the tubulin compartment is the least gravity-sensitive cytoskeleton structure.

The transmission of a mechanical signal through integrins is associated with the clustering of these receptors and the formation of focal contacts. Several studies have shown changes in the molecular structure of the focal contacts due to the cytoskeleton–ECM association under microgravity [39,40,42]. The transcription profile of integrin-encoding genes and the expression of the corresponding molecules have been shown to significantly change. A downregulation of some genes encoding focal contact proteins, including *FAK, DOCK1* and *PTEN*, was demonstrated, while *CAV1* (caveolin) and *RB2/p130* were upregulated [42,43]. Under simulated microgravity, a decreased number of focal adhesion sites on cells and their redistribution to the area above the nucleus were observed after 30 min and reached their maximum at 48–120 h. The changes over time were quite consistent with the above-mentioned F-actin stress fibrils remodeling [26]. The decreased numbers and sizes of focal contacts may adversely affect the ability of cells to adhere, migrate, and maintain their viability [44,45,46,47]. These findings suggest that transient rearrangements of the cytoskeleton and associated surface receptors may be the primary events of cell adaptation to microgravity since they are manifested very shortly after the gravitational field alteration.

More recently, a specialized structure known as the linker of nucleoskeleton and cytoskeleton (LINC) was found in the nuclear lamina. LINC provides a functional interaction between the supporting structures of the cytoplasmic and nuclear compartments [48]. Therefore, the nuclear lamina acts as a mechanosensitive element that regulates both the biochemical and physical nucleo- and cytoskeleton linking [49,50,51].

Unlike the cytoskeletal and transmembrane compartments, the study of microgravity effects in the ECM is at an early stage. This may be due to methodological difficulties, such as the molecular diversity of the ECM and the complexity of isolation and analysis. At the same time, the structural uniformity of the ECM in various tissues and organs can augment the function of the ECM as an integral gravity sensor. Before proceeding to a more detailed discussion of the currently available data on the fate of ECM under real and simulated microgravity, it seems appropriate to give a brief summary of the contemporary views on the ECM.

## 3. Extracellular Matrix Composition and Tensegrity

For decades, ECM was considered to play a structural role only in tissue shape maintenance under mechanical loading and physical support for cell adhesion and migration. Recent studies have shown that the ECM also plays an important instructive role, providing biochemical and biomechanical signals that affect cell activity, including migration, adhesion, phenotypic modulation, and survival. With regard to the subject of this review, the question arises whether ECM can act as a mechanosensitive detector, triggering the tissue response circuit to mechanical impact or its deprivation under microgravity.

According to modern concepts, ECM not only forms a three-dimensional molecular network around cells, thus providing physical maintenance of tissue integrity and elasticity but is also a flexible structure constantly being remodeled through protease/antiprotease activity to control tissue homeostasis [52,53].

Recently, the “matrisome” term has been proposed to describe ECM and associated molecules [54]. This includes the core matrisome, matrisome-associated molecules, and matrisome-affiliated molecules.

*Core matrisome* ECM is presented by collagens, glycoproteins, and proteoglycans. Collagens are the core structural proteins of ECM and are classified as fibrillar (collagen types I–III, V, and XI) and nonfibrillar [55]. The contribution of collagen fibers to the overall tissue structural integrity depends on the fiber density, orientation, cross-linking, pre-stressing, and interaction with other matrix components [56]. Being the most abundant proteins in mammals, collagen fibers provide tissue stiffness (the degree of tension changes under stress) and strength (maximum stress at break) that limits their flexibility. Having a relatively short half-life, they are not subject to mechanical fatigue [56].

Glycoproteins, such as elastins, laminins, fibronectins, thrombospondins, tenascins, and nidogen, possess a variety of functions due to their various molecule-binding domains [57,58]. In addition to their involvement in the formation of the ECM structure (due to binding to other ECM components), glycoproteins play an essential role in ECM–cell interaction (glycoprotein RGD domains are ligands for integrins) [59].

Elastic fibrils play a key role in providing tissue extensibility and resilience (the ability to recoil upon unloading). The synthesis of elastin mainly occurs during the fetal period [60]. After birth, elastogenesis is attenuated and disappears at puberty [61]. The neo-synthesis of elastin is low or inexistent [62]. Thus, elastic fibrils are deposited and mature for decades and are the most biologically, chemically, and thermally stable ECM components, providing “mechanical memory” [63]. In this regard, the mechanical damage or proteolytic degradation to which they are subjected can result in irreversible changes in tissue shapes and functions [56].

Proteoglycans (such as aggrecan, versican, perlecan, and decorin) form a “brush” with glycosaminoglycans (GAG) side chains attached to a core protein. Hyaluronic acid is unique among other GAGs since it is synthesized on the cell membrane and does not bind to core protein but can noncovalently bind to other proteoglycans. Proteoglycans are commonly classified as intracellular, cell surface-bound, pericellular, and extracellular and provide tissue hydration by retaining water [55]. Extracellular proteoglycans and hyaluronic acid fill the spaces between cells, being localized throughout the collagen fibers forming the so-called ground substance. The entire intercellular transport passes through the ground substance, and growth factor deposition/release occurs therein. This mixed gel-like complex of GAGs, proteoglycans, and glycoproteins (mainly laminin and fibronectin) has a very high ability to retain water. This property ensures tissue resistance against compression. In addition, the ground substance provides the correct stacking of tropocollagen molecules in fibrils and fibril packaging in collagen fibers that are also in charge of pressure resistance [55].

*Matrisome-associated molecules* include ECM remodeling regulators, and secreted and deposited factors. ECM degrading enzymes (matrix metalloproteinases, serine proteases, and cathepsins) can directly degrade matrix proteins to produce signaling molecules (matrikines) or release deposited growth factors. Transglutaminases, lysyl oxidases, and prolyl hydroxylases provide ECM maturation in extracellular spaces, thus forming a tissue-specific microenvironment [63]. The balance of proteases and relevant inhibitors is important to maintain the homeostasis of adult tissues under normal development. Their misbalance can result in various pathological processes, including tumor progression [1].

ECM structures are able to bind and deposit various biologically active mediators, growth factors, cytokines, and morphogens. These mediators can be released in response to microenvironmental signals [64]. Various combinations of matrix molecules have been found to bind and retain growth factors such as TGF-β, hepatocyte growth factor (HGF), insulin-like growth factor (IGF), and platelet-derived growth factor (PDGF) [2]. This makes it possible to stabilize and increase the local levels of mediators, guide their diffusion through the matrix, and protect against proteolytic degradation [65,66]. In addition, cells produce fibronectin, proteoglycans, and several other ECM macromolecules in a soluble form. These macromolecules can be considered as a special group of local mediators, affecting the neighboring cells only [65]. Unlike other local soluble mediators, they quickly lose motility and therefore cannot diffuse from their secretion sites. In the extracellular space, these molecules join the ECM to form an insoluble network. The products of glycoprotein fragmentation, matrikines, with their regulatory functions different from those of the original full-sized molecules, are retained in the ECM as well [67,68].

*Matrisome-affiliated molecules* include cytokines, mucins, secreted lectins, galectins, semaphorins, and plexins that are not constitutive components of ECM but may be associated with it on a functional request [69].

Therefore, the ECM determines the structural integrity in the extracellular space by presenting cell adhesion sites, thereby limiting or stimulating cell movement and controlling the diffusion of morphogens. Therefore, the ECM can be considered as a so-called “tissue code or language” since its rigidity, elasticity, or hydration determine the mechanical properties of the tissue [70]. In the implementation of ECM tensegrity, elastic structures act as a tensional element, while individual collagen fibers as well as ground substance are compression-resistant elements [11]. The extracellular tensegrity network is considered as a solid-state regulatory system for all cell functions responsible for alterations in genes and proteins, as well as for changes in cell shapes and motility [11,71,72].

The interaction of cellular and extracellular tensegrity systems supports tissue mechanical homeostasis. To provide this, cells have to use feedback mechanisms that detect changes in the ECM and restore values to their steady-state levels. For example, an acute increase in stiffness should trigger mechanisms that make the ECM more flexible, whereas an acute reduction in stiffness should trigger pathways that increase stiffness.

There are several questions on whether and how the major mechanical properties of compression and tension of the existing ECM change upon gravity deprivation. How can this affect the properties of the de novo synthesized matrix?

## 4. Extracellular Matrix and Microgravity

Gravity is a universal mechanical stimulus that determines the mechanical homeostasis of tissues on the Earth. Accordingly, the ECM tensegrity stabilizing elements, i.e., tension and compression, will be affected by gravitational unloading at all structural levels of the body. From this point of view, the skeletal system is most sensitive due to a significant amount of ECM.

Normal bone tissue homeostasis is based on the dynamic balance between two closely related physiological processes: the formation and degradation of the bone matrix [73]. This ensures the functional adaptation of bone tissue depending on the requirements of the “external mechanical field”, including the levels of loading support, as has been shown in vivo [74,75,76].

With the beginning of human exploration of space, the issue of microgravity effects on the bone system has become particularly important. To date, it is known that bone mass may decrease due to the attenuation of mechanical impulses with the lack of mechanical loading (hypokinesia, hypodynamia, or immobilization) [77]. It was assumed to occur due to the dysregulation of concomitant adaptive bone tissue remodeling. Similar deformations were supposed to occur under microgravity [4,74,78]. The changes in mineral metabolism and human bone tissue state during space missions have fully justified these theoretical prerequisites. The data set of the detected physiological changes included the loss of total bone mass, demineralization of bones bearing an axial support loading on the Earth, a negative calcium balance, and increased bone resorption markers in blood and urine [4,78,79]. These data convincingly indicate that atrophic bone tissue rearrangements do occur under microgravity, with their severity largely depending on the bone location relative to the gravity vector [4,80,81,82]. Obviously, this fact can significantly limit the duration of a human stay in space due to the potential risk of osteoporosis and be followed by adverse consequences upon the return to the gravity of Earth.

During manned space flights, special attention is paid to countermeasures to reduce the adverse effects of microgravity, which can attenuate the severity of changes in the skeletal system. Therefore, the examination of the effects of real and simulated microgravity in experimental animals is of particular interest because of the absence of preventive measures.

The opportunities for aboard animal experiments are extremely limited. Only a few of such studies have obtained data on ECM and its components. In rats, after 18–22 days at the Bion-M1 Cosmos biosatellite program, a reduced mineralization of bone tissue, an inhibition of periosteal and endosteal remodeling in trabecular bones and the vertebrae, a decreased number of osteoblasts, and an unchanged number of osteoclasts were observed [83,84,85,86,87,88].

A reduced level of collagen type I and the appearance of collagen type III typical of embryonic tissues and early stages of inflammation and healing were found in the bone tissue [89]. In STS-1 and STS-2 experiments, after 9–14 days of zero gravity, mice had initial signs of tibial osteopenia and inhibition of bone growth in length [87]. Downregulation of genes encoding bone tissue proteins, osteonectin, osteocalcin, and collagen type I in femoral bone cells was observed [90]. The exposure of murine embryonic bone tissue culture at the Kosmos-2229 biosatellite was followed by the rate reduction in mineralization with a simultaneous increase in the tissue resorption rate [91].

After a 30-day flight at the Bion-M1 biosatellite, significant alterations were found in murine cartilage tissues: a decrease in articular cartilage proteoglycans associated with the downregulation of genes encoding mechano-responsive and structural cartilage matrix proteins, fibromodulin (*FMOD*), osteoglycin (*OGN*), clusterin (*CLU*), decorin (*DCN*), collagen X (*COL10A1*), trombospondin-4 (*TSP4*), and cartilage oligomeric protein (*COMP*). In contrast, no such disturbances were found in the sternal cartilage. The authors emphasized that it was the weight-bearing articular cartilage and not the minimally loaded sternal fibrocartilage that was adversely affected by microgravity [92].

Aboard the ISS, live imaging of medaka fish demonstrated an excessive DsRed-osteocalcin glycoprotein fluorescence compared to the ground control in pharyngeal bone osteoblasts at flight day 1. At flight days 5 and 8, the increased fluorescent signal persisted. Post-flight HiSeq global gene analysis found upregulation of genes encoding matrix proteins of osteoblasts: *OCN* (osteocalcin) and collagen type X (*COL10A1*). Based on the above, the authors concluded that the transcriptional response to microgravity developed very rapidly [93].

In 2021, Fu et al. published the results of a meta-analysis of available data on space flight bone tissue changes in rodents and primates. All experiments reported a decreased bone mass, more significant in trabecular bones compared to the cortical bones. The bones that were weight bearing under normal gravity were affected to a greater extent than the minimally loaded ones. Changes in the lower extremity bones were much more pronounced than that in the upper ones. The rate of trabecular bone mass loss was almost independent of the flight duration and was −1.7% a day. In comparison, the authors indicated that this parameter was much lower for astronauts (0.7–2.7% per month) due to the countermeasures undertaken. Osteoblast indices in the rodent trabecular bone were significantly lower than in cortical bones, while the numbers of osteoclasts showed no changes in rats and varied in mice. Based on the meta-analysis, the authors concluded that microgravity causes a bone tissue deficiency in rodents and primates, which may be associated with the decreased activity of osteogenic cells [94].

The hind limb suspension model is the most common one for ground-based microgravity simulation experiments. In specially equipped cages, rodents are placed in such a way that the hind limbs are deprived of support, while the animals can move freely on their front paws and have unlimited access to water and food [95]. Rodent experiments of various durations (up to 28 weeks) demonstrated a significant decrease in the bone mass in the trabecular parts of the femur and tibia, similar to the effects of inflight experiments (1.1–3.5% a day). In addition, similar to during the flights, the changes in the trabecular parts of bones were more significant than in the cortical ones.

Local bone mass losses under mechanical load deficiency or microgravity suggest that a mechanical signal (or its lack) can also be perceived at the cell level. The stromal lineage cells represented both by differentiated and progenitor elements can be involved in the process.

In vitro, experiments of various durations using osteogenic precursors have been performed in several space flights. Both at the unmanned Foton-10 satellite [96] and in the manned STS-54 [97] and STS-59 [98] missions, a downregulation of *OCN*-encoded glycoprotein osteocalcin was detected from flight day 5 to day 12. In addition, experiments at Foton-10 and STS-59 demonstrated a decreased transcription of *COL1A2* (collagen type I). With increased flight duration, after 17 days on board STS-80, the expression of a number of osteogenesis-related genes (*ALPL*, alkaline phosphatase; *ON*, osteonectin; and *OCN*, osteocalcin) in human fetal osteoblasts showed no differences from ground controls [99].

Therefore, inflight observations are the basis to believe that microgravity adversely affects the transcription of genes encoding ECM proteins during up to 14-day flights. The increased fluorescent signal of DsRed-osteocalcin described in medaka fish may indicate the post-translational effects of microgravity [93].

To study the effects of gravity deprivation on cells on the Earth, various simulating equipment has been developed. These devices include 2D and 3D clinostats, a random positioning machine (RPM), and rotary wall vessels (RWV) [100]. The common feature of all ground-based devices is the randomization of cell position relative to the gravity vector. Such equipment provides the opportunity to elucidate the mechanisms of the altered hypogravity impact on cells and to adapt methodological approaches prior to their use in space missions.

Gravity vector randomization studies using 2D/3D clinostats and RPMs detected multidirectional changes in the transcription of genes encoding structural proteins, as well as of ECM-associated and affiliated molecules. Buken et al.(2019), after a three-day fibroblast RPM exposure, described an increase in *COL4A5* and transcription/translation of the major ECM glycoprotein, fibronectin *FN*/FN. In MC3T3-E2 osteoblasts, the transcription of genes encoding enzymes that provide extracellular post-translational modification of collagen fibers, *PLOD1* and *PLOD2*, and the functional activity of these enzymes were increased during clinorotation [101]. After 7 days of 2D clinorotation, an increase in the expression of *COL1* and *COL3* was observed in MSCs [102]. On the other hand, there is evidence of a decrease in the expression of *COL1A* and *FBN1* (fibrillin) after 7 day’s aclinorotation [102,103]. Regardless of the model and exposure time, a downregulation of *OMD* (osteomodulin) that regulates adhesion was shown in the preosteoblasts [26,104]. In addition to changes in structural proteins, a decreased transcription of genes encoding molecules associated with ECM metabolism, transcription factors, *cbfa1/RUNX2*, and growth factors *BMP3, FGF4, GDF2*, and *GDF3* was observed [26,105].

The available data suggest the existence of complex time-dependent changes in the transcriptional activity of matrix-associated genes. After 5 days at RPM, the transcription of *COL1A1*, *COL2A1*, and *COL9A1* was downregulated in bone marrow MSCs. After 10 days, no difference was found, and after 20 days, a significant upregulation compared to the static control was observed. At the same time, after 10 and 20 days of exposure, the expression of *OMD* was reduced, and the expression of *OCN* had no differences vs. static control [26]. According to Ratushnyy and Buravkova (2017), after a 96-h of adipose MSC exposure at RPM, upregulation of *COL12A1*, *COL15A1*, *COL16A1*, *COL1A1*, *COL5A1*, and *COL8A1* and the glycoproteins *THBS1*, *THBS2*, *THBS3*, *LAMA*, *SPARC*, *TNC*, *VCAN*, and *VTN* was observed. With a longer exposure (10 days), the number of ECM genes with an altered transcription decreased. An increase was found only in *COL11A1*, L*AMB3*, and *TN* [43].

In addition to the stromal cell transcriptional activity, several studies (cited above) analyzed the ECM features per se. Zhivodernikov et al. (2020) described a decrease in collagenous and an increase in noncollagenous proteins in MSC ECM after a 10-day RPM exposure [106]. After 12 days of MSC clinorotation, a decreased mineralization of the ECM was detected [107]. A similar effect was found after a 20-day RPM exposure of osteo-induced MSCs [26]. Interestingly, a long-term (20 days) RPM microgravity simulation had different effects on the mineralization efficacy of the ECM secreted by stromal cells of different commitments: calcium deposition was attenuated in osteo-committed MSCs, and, on the contrary, it was increased in osteoblasts [26].

The data from several studies clearly demonstrated the significant alteration in the transcription levels of matrisome-related genes in stromal lineage cells. This list includes genes encoding core matrisome compression-resistant proteins such as collagens (*COL*), laminins (*LAMA*, *LAMB*), as well as glycoproteins: trombospondins (*THBS*), fibrillin (*FBN*), tenascin (*TNC*), vitronectin (*VTN*), etc. In addition, the genes of the ECM-remodeling protein encoding lysyl hydroxylases (*PLOD1*, *PLOD2*) also changed transcription. Above glycoproteins, so-called matricellular proteins are involved in the regulation of tissue development, function, and regeneration by controlling cellular response. These molecules act as a depot of growth factors and other bioactive mediators. The retention/release of these molecules governs cell adhesion, migration, proliferation, differentiation, etc. [6]. The complex changes in transcription/production of matrisome components under microgravity no doubts modifies not only the gravisensitivity of cells, but their functions as well. Under short-term real or simulated microgravity, multidirectional changes in the transcriptional activity of ECM-associated genes were found to provide the adaptation of stromal lineage cells with the above impact. In general, the direction of these changes is independent of the commitment level of stromal precursors. The few observations regarding ECM features make it possible to conclude that the ECM content and its mineralization decrease as a result of microgravity.

## 5. Concluding Remarks

**Figure 2:**
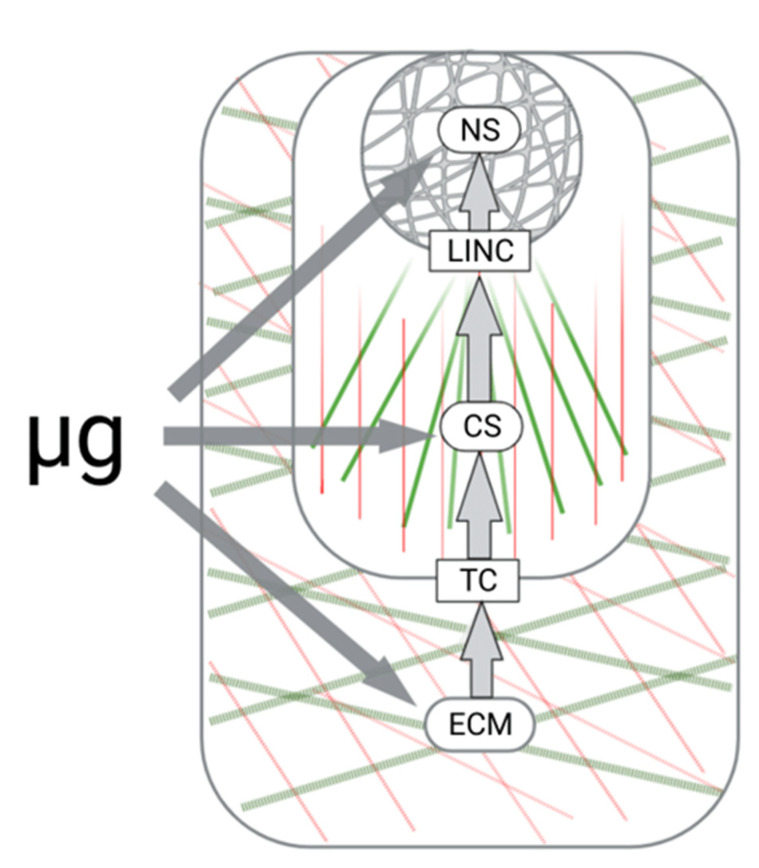
Mechanoreception check-points and microgravity. Both extracellular and cellular compartments of the mechanosensitive functional unit are targets for gravity deprivation signals. The extracellular matrix (ECM), cytoskeleton (SC), and nucleoskeleton (NS) structures may be considered as gravity sensors. Specialized molecular hubs, transmembrane complexes (TCs), and the linkers of nucleoskeleton and cytoskeleton (LINC), provide the conversion of altered gravity signals into the nucleus mediating microgravity-affected mechanotransduction. Compression-resistant ECM and CS elements are colored in green. Tensional elements are colored in red.

The lack of gravitational loading turned out to be the most important challenge that humanity faced after leaving the surface of the Earth. Microgravity determines the quality of life of humans and other mammals aboard spacecraft, changing the biomechanics of the body at all levels: at the macroscale level (e.g., organs and tissues), microscale level (e.g., individual cells), and at the nanoscale level (e.g., molecular complexes or individual proteins) [108]. When applied to cell niches, these are feedback loops involving extracellular, transmembrane, cellular, and nuclear compartments. From the point of view of mechanobiology, all these compartments are mechanosensitive, and their interaction determines the execution of mechanotransduction, thus providing adaptation to changes in the mechanical field. It is obvious that the lack of a permanent gravitational load significantly modulates all structural compartments of mechanoreception and mechanotransduction (Figure 2).

In space flights, as well as in ground-based experiments, the coupling of mechanical incoming and biochemical outgoing signals due to the interaction of cellular tensegrity system and integrin-based focal adhesion sites (mechanotransduction) have been studied in detail. Here, we attempted to highlight data on the changes in the extracellular tensegrity system under gravity deprivation. The papers related to stromal and skeleton tissues as the most affected ones during space flights were selected for analysis. It turned out that most available data were from the analysis of the transcriptional/translational activity of stromal lineage cells, whereas observations on the changes in the ECM under altered gravitational load were few [102,106]. The loss of bone and muscle mass, undoubtedly associated with changes in the connective tissue properties of ECM, is one of the most noticeable adverse effects of microgravity [4,80]. These facts support the urgent need to expand and structure the knowledge on ECM mechanosensitivity in the context of gravity deprivation.
